# Effects of predation risk on egg steroid profiles across multiple populations of threespine stickleback

**DOI:** 10.1038/s41598-020-61412-5

**Published:** 2020-03-23

**Authors:** Katie E. McGhee, Ryan T. Paitz, John A. Baker, Susan A. Foster, Alison M. Bell

**Affiliations:** 1 Department of Biology, The University of the South, Sewanee, TN USA; 2 School of Biological Sciences, Illinois State University, Normal, IL USA; 3 Department of Biology, Clark University, Worcester, MA USA; 4 School of Integrative Biology, Program in Neuroscience, Program in Ecology, Evolution and Conservation Biology, Carl R. Woese Institute for Genomic Biology, University of Illinois, Urbana Champaign, Urbana, IL USA

**Keywords:** Ecology, Evolution, Endocrinology

## Abstract

Predation often has consistent effects on prey behavior and morphology, but whether the physiological mechanisms underlying these effects show similarly consistent patterns across different populations remains an open question. In vertebrates, predation risk activates the hypothalamic-pituitary-adrenal (HPA) axis, and there is growing evidence that activation of the maternal HPA axis can have intergenerational consequences via, for example, maternally-derived steroids in eggs. Here, we investigated how predation risk affects a suite of maternally-derived steroids in threespine stickleback eggs across nine Alaskan lakes that vary in whether predatory trout are absent, native, or have been stocked within the last 25 years. Using liquid chromatography coupled with mass spectroscopy (LC-MS/MS), we detected 20 steroids within unfertilized eggs. Factor analysis suggests that steroids covary within and across steroid classes (i.e. glucocorticoids, progestogens, sex steroids), emphasizing the modularity and interconnectedness of the endocrine response. Surprisingly, egg steroid profiles were not significantly associated with predator regime, although they were more variable when predators were absent compared to when predators were present, with either native or stocked trout. Despite being the most abundant steroid, cortisol was not consistently associated with predation regime. Thus, while predators can affect steroids in adults, including mothers, the link between maternal stress and embryonic development is more complex than a simple one-to-one relationship between the population-level predation risk experienced by mothers and the steroids mothers transfer to their eggs.

## Introduction

Predation is a potent force that shapes phenotypes over evolutionary and developmental time^1,2^. A major question in evolutionary biology is the extent to which selective pressures such as predation pressure have predictable effects on prey phenotypes and the role of natural selection in shaping consistent patterns of plasticity and/or the independent evolution of similar traits in closely related lineages (i.e. parallelism^3–6^). Examining whether different populations of prey respond to predators in the same manner has offered insights into this question^7^. For example, traits that protect prey and/or enable quick escape from predators often show evidence of parallelism across replicate populations with similar predation regimes (e.g. morphology^7,8^; antipredator behavior^9,10^). Predation pressure has also been shown to affect life history traits (e.g. age and size at maturity, offspring size and number), often in a parallel manner across populations with similar predation regimes^11,12^. Despite the many studies comparing morphology, behavior, and life history traits among populations that differ in predation risk, less is known about the predictability of changes in physiological mechanisms underlying these phenotypic responses to predation pressure.

The physiological response to acute and chronic stressors such as predation risk is highly conserved across vertebrates^13,14^ and largely mediated by the hypothalamic-pituitary-adrenal axis (HPA - mammals, reptiles and birds^13^) or the hypothalamic-pituitary-interrenal axis (HPI – fish^15^). The glucocorticoid steroid hormones produced by the HPA (cortisol in most mammals and fish; corticosterone in birds, rodents, reptiles and amphibians) are likely to be especially responsive to predation risk^2,14^. For example, encounters with predators increase short-term levels of cortisol in fishes^16^, corticosterone in birds^17^, and fecal cortisol metabolites in mammals^18^. Similarly, when predators vary seasonally and/or spatially over weeks and months, prey show elevated glucocorticoid levels that correspond to predation risk^18,19^ (but see^20,21^), potentially due to frequent encounters with predators. When predation risk is stable over generations, baseline levels and stress responsiveness can also be affected. For example, individuals from consistently high-predation risk populations have lower levels of cortisol after a stressor compared to individuals from low-predation risk^21,22^. How the evolutionary history of predation risk affects the physiological response to stress however, remains surprisingly understudied^21,23^.

While the glucocorticoid mediated stress response modulates metabolic and behavioral changes that enable animals to cope with stressors^24^, there are often other physiological consequences^25^. Fundamentally, steroid hormones are biochemically related to one another, with all steroids initially derived from cholesterol and linked via various enzymatic conversions^26^. Thus, physiological changes in response to predation pressure will involve multiple steroid hormones to orchestrate the phenotypic response to predation risk. For example, activation of the HPA axis can decrease the production of sex steroids such as progesterone and estradiol by the hypothalamic-pituitary-gonadal (HPG) axis^25,27,28^. This link between the HPA and HPG axes creates a situation in which stressors are likely to influence levels of multiple steroids, not just glucocorticoids^29^. Furthermore, when predators encounter mothers provisioning eggs or gestating offspring, the hormonal consequences of predation risk have the potential to affect future generations through the hormones mothers transfer to offspring^30,31^.

Exposing females to stressors, such as predators, during reproduction can have long lasting effects on their offspring across a broad range of taxa^30,32–34^. Some of these effects are thought to be mediated by offspring exposure to increased levels of maternal glucocorticoids during development^31,34–37^, but not all maternal stress effects are mediated by embryonic exposure to maternal glucocorticoids^38^. Exposing females to stressors such as increased predation risk can also influence the amount of sex steroids transferred to eggs^39^. Taken together, increases in predation risk have the potential to not only elicit changes in glucocorticoid and sex steroid levels in a female but also to alter the levels of these steroids within her eggs and the developmental environment of her offspring.

The threespine stickleback (*Gasterosteus aculeatus*) adaptive radiation offers an opportunity to evaluate the extent of parallel changes in response to predation because postglacial freshwater populations within a region are all derived from a common marine ancestral type. Freshwater populations in disparate river drainages have been colonized independently and may thus be considered independent replicates^40^. Work in stickleback has drawn attention to parallel evolutionary changes in morphological and behavioral defenses (reviewed in^8,41^; note that parallel, rather than convergent, is the term used in this system^42^ and thus the term we use). These changes can be surprisingly rapid^8,41,43^. For example, the loss of armor plating after colonization of a freshwater habitat has been shown to occur in less than 10 years^44^. Here we examine whether these patterns extend to the physiological response and the potential maternal effects due to predation risk.

Specifically, we examined how predation risk affects maternally derived steroids in eggs, and thus the endocrine state experienced by developing offspring, in threespine stickleback. We assessed whether egg steroid content is associated with predation regime among three sets of freshwater lakes: those devoid of piscine predators for ca 6,000 generations (“predatory fish absent”), those exposed to rainbow trout for ca 6,000 generations (“native predatory fish”) and those exposed to rainbow trout for 25 or fewer generations (“stocked predatory fish”), including n = 3 lakes per predator regime. Although we do not have phenotypic or behavioral information on these populations, substantial previous work on stickleback has demonstrated the strong influence of predation on many phenotypic traits^8,10,41,43,44^.

Our sampling design allowed us to test three hypotheses. First, we examined whether the steroids that mothers deposit into their eggs show similar patterns in response to predation pressure. Given the important role of glucocorticoids in mediating the response to stressors, including predation, we predicted that populations with predatory trout would have higher egg glucocorticoid content in their eggs compared to populations without predatory trout. These elevated levels of glucocorticoids in eggs of females from high predation risk populations could be due to (1) encounters with predators resulting in acute stress responses during egg formation, (2) encounters with predators leading to the elevation of baseline glucocorticoids in females, and (3) evolved differences in baseline levels of glucocorticoids. Second, we examined whether patterns of maternal steroid provisioning in eggs of “stocked” populations that have experienced ~25 years with predatory trout resemble those of “native” predatory fish populations or those of populations in which predatory fish are “absent”. Note that morphological changes due to ecological conditions can occur in fewer than 10 years in stickleback^44^. Finally, we examined whether the steroids within eggs change as a coordinated package or each independently in response to predation risk. We expected glucocorticoids to be most responsive to variation in predation pressure, but given the interconnected nature of the HPA and HPG axes in vertebrates, we hypothesized that predation risk would simultaneously influence levels of numerous steroids within eggs. Therefore, we used liquid chromatography coupled with mass spectroscopy (LC-MS/MS) to simultaneously measure 21 steroids in individual egg clutches^45,46^. We then analyzed the egg steroid content data in a multivariate fashion in order to assess whether the components of the egg steroid profile change in concert or independently.

## Materials and Methods

### Study populations

Eggs were collected from females in nine lacustrine populations of threespine stickleback in the Matanuska-Susitna Valley of southcentral Alaska. This region was heavily glaciated until the most recent recession began ca. 12000 years ago providing a maximum age for freshwater populations in the region^40^. Oceanic stickleback colonized newly formed freshwater habitats giving rise to a remarkable adaptive radiation^8,40^. To examine how predators influence levels of maternally derived steroids in stickleback eggs, we took advantage of historical information on piscivorous fish in the nine study lakes. Three of the study lakes possess native populations of rainbow trout (*Onchorynchus mykiss*) (native: Beaver House, Kashwitna, South Rolly), three are historically devoid of piscivorous fish (absent: High Ridge, Jean, Whale), and three were devoid of piscivorous fish until stocking with rainbow trout began in 1992 (stocked: Bear Paw, Dawn, Vera) (see Table S1 for lake details). Recent studies have advocated the use of a geometric definition of (non)parallelism^3,6^. This method allows comparison of angles among phenotypic trajectories to examine repeated evolutionary patterns, but paired ancestral and descendant populations are necessary to estimate vectors^3,6,47^. Our design is unpaired^48^ without known ancestral populations for each lake, and thus we were not able to use this geometric method.

### Egg collection

Between June 4 and 7, 2013 gravid female were collected from these lakes using unbaited minnow traps set for approximately 24 hr (range 18–26 hr) and eggs from 9–10 ovulated females in each population were extruded by gently squeezing the abdomen (N = 89 total clutches; 10 clutches from each Lake except for Whale Lake with absent predators where 9 clutches were collected). The eggs were immediately frozen on dry ice until transported to a freezer. Eggs were shipped overnight to the University of Illinois on dry ice and then were held at −20 C until steroid analysis.

All methods were performed in accordance with relevant guidelines and regulations and all experimental protocols were approved by the Animal Care and Use Committee at University of Illinois (no. 12118).

### Steroid quantification via LC/MS/MS

The use of liquid chromatography coupled with mass spectroscopy (LC-MS/MS) has become an essential technique for quantifying steroids^45^. While often not as sensitive as traditional antibody-based techniques such as radioimmunoassay^49^, LC-MS/MS eliminates issues related to antibody cross-reactivity and can simultaneously quantify numerous steroids within a biological sample^46^. Steroid standards were purchased from Steraloids, Inc. (Newport, RI, USA) and prepared in methanol at a concentration of 1 mg/ml. Overall, 21 different steroids were quantified (steroid quantification details in Table S2) in order to achieve a holistic profile of steroid produced by the HPI and HPG axis. This resulted in levels of multiple glucocorticoids (n = 9), progestogens (n = 5), androgens (n = 5), and estrogens (n = 2) being characterized.

To prepare eggs for steroid quantification, eggs were thawed, weighed, and homogenized with a Potter-Elvehjem homogenizer. Steroids were first extracted with methanol^50^ by adding 2 ml of 100% methanol to sample homogenate and vortexing for 60 sec. Samples were stored at −20 °C overnight to precipitate neutral lipids and proteins. The samples were then spun at 2000 rpm at 0 °C for 20 min and the supernatant was poured to a 50 mL conical vial. A fresh 2 ml of methanol was added to the remaining pellet and the extraction process was repeated with the methanol supernatant being added to the first 2 ml of methanol extract. This 4 ml of methanol was diluted with 46 ml of MilliQ water (Millipore, Bedford, MA, USA) to bring the final volume up to 50 ml that was subsequently subjected to solid phase extraction of the steroids^51^. Additionally, standards were prepared to estimate the efficiency of solid phase extraction by adding 50 ng of each steroid to 4 ml of methanol and adding 46 ml of water.

Solid phase extraction was carried out with C18 Sep-pak cartridges (Waters, Ltd., Watford, UK) that were charged with 5 ml methanol and rinsed with 5 ml of water before the sample was loaded^52,53^. Samples were loaded under vacuum pressure at a drip rate of approximately 2 ml/min. Once samples had been pulled through, cartridges were rinsed with 5 ml of water. Steroids were finally eluted with diethyl ether and dried under nitrogen gas. Dried samples were submitted to the Metabolomics Lab of Roy J. Carver Biotechnology Center, University of Illinois at Urbana-Champaign for quantification.

Samples were analyzed with a 5500 QTRAP LC/MS/MS system (AB Sciex, Foster City, CA). The 1200 series HPLC system (Agilent Technologies, Santa Clara, CA) includes a degasser, an autosampler, and a binary pump as described in^53^. The LC separation was performed on a Phenomenex C6 Phenyl column (2.0 × 100 mm, 3 μm.) with mobile phase A (0.1% formic acid in water) and mobile phase B (0.1% formic acid in acetonitrile). The flow rate was 0.25 mL/min. The linear gradient was as follows: 0–1 min, 80%A; 10 min, 65%A; 15 min, 50%A; 20 min, 40%A; 25 min, 30%A; 30 min, 20%A; 30.5–38 min, 80%A. The autosampler was set at 5 °C. The injection volume was 5 μL. Mass spectra were acquired under positive electrospray ionization (ESI) with the ion spray voltage of 5500 V. The source temperature was 500 °C. The curtain gas, ion source gas 1, and ion source gas 2 were 36 psi, 50 psi, and 65 psi, respectively. Multiple reaction monitoring (MRM) was used to measure steroids (Table S2). D9-progesterone (from m/z 324.1 to m/z 100.1) was used as an internal standard to control for inter-sample variation.

### Statistical analyses

All steroids were converted to concentrations based on the wet mass of the clutch (ng of steroid per gram of eggs) after correcting for the average extraction efficiency (see Table S2 for % recovery and coefficients of variation for all steroids). We used Spearman correlations to explore how the measured steroids were related to one another.

To examine whether presence of piscivorous trout (absent, native, stocked) affects the steroids mothers transfer to their egg clutches, we used a permutational (non-parametric) MANOVA approach. First, we standardized all steroid concentrations using the ‘log’ standardization (*decostand* command) where x-values of zero are left zeros and logb(x) + 1 for all x-values greater than zero (b is the base of the logarithm), and specifying that standardization was within steroids (i.e. columns, MARGINS = 2)^54,55^. Second, we created a dissimilarity matrix on the standardized data of the steroids specifying Euclidean distances, similar to dissimilarity matrices used to compare communities in species abundances (R package vegan: *vegdist* command). Third, we compared the dissimilarity matrices among predation regime (absent, native, stocked) with Lake nested within Predation regime using a permutational (non-parametric) MANOVA approach with the program *adonis* (R package vegan^54,55^). We specified a permutation regime in which clutches/samples were permutated within Lake and Lake within Predation regime (Hank Stevens, personal communication). P-values were estimated with 10000 permutations and we specified “Euclidean distance” for the distance matrix. Although we examined the steroid concentrations (ng/g), it is unknown whether the concentration of different steroids vary with clutch size since there may be an endocrinological cue to increased clutch mass. For example, increased maternal production of progesterone could result in larger clutches that also have higher amounts of these steroids. Thus, we included wet clutch mass as a covariate in the MANOVA analyses of all steroids.

This permutational MANOVA approach requires an equal number of samples within each category to ensure appropriate permutations. Since one of our Lakes (Whale – absent) only had nine clutches, we created a 10^th^ sample by averaging the traits from the existing nine samples from this Lake, thus making 10 samples per Lake and a total of 90 samples. This n = 90 dataset was used in the permutational MANOVA as well as in the factor analysis. To ensure this did not affect our conclusions, we also conducted all of the same analyses with a single random sample removed from each lake other than Whale, thus making 9 samples per Lake and a total of 81 samples.

Because the different steroids are related to one another within clutches and are likely to vary in a coordinated fashion, we used factor analysis with varimax rotation and maximum likelihood to extract underlying factors from the concentrations of the measured steroids (R package psych^56,57^). The number of factors to extract was assessed from parallel analysis and a scree plot. Using these factors, we examined the relationships among steroids as well as whether predation regimes differed in their factor scores. Specifically, we examined whether these extracted factors differed among predation regime using a similar permutational MANOVA analysis to that for the entire steroid profile (described above), with wet clutch mass included as a covariate. We also explored whether the variation in these extracted factors differed among predation regimes using a Fligner-Killeen test of homogeneity of variances. We used Spearman correlations to examine the relationships among these factors and clutch mass across predation regimes.

Because the factors were extracted from the entire data set, the relationships among steroids are not able to vary independently among the different predation regimes. While the permutational MANOVA examining the distance matrix among all measured steroids does allow these relationships to freely vary, we wanted to explore whether the make-up of the factors themselves might differ among predation regimes. We used the Common Principal Component Analysis program^58^ to compare the covariance matrices of the hormones (after the decostand standardization described above) for each predation regime, specifying the extraction of three principal components. We took a model building approach^58^ and focused on the model with the lowest AIC. Note that ‘Lake’ cannot be specified in these analyses, so we interpret these analyses with caution.

In separate mixed models, we examined how the concentration of cortisol (ng/g) in clutches, as well as the wet clutch mass, varied with predation regime including ‘Lake’ nested within ‘Predation regime’ (R package nlme^59^). To appropriately test for the effect of predation regime, we used the Satterthwaite degrees of freedom estimation (R package lmerTest^60^). Cortisol concentration was natural log-transformed based on the residuals while wet clutch mass was left untransformed. Wet clutch mass was included as a covariate in the cortisol analysis.

All analyses were conducted in R, version 3.4.4^61^. Data was manipulated with R package dplyr^62^ and figures were made in R packages ggplot2^63^, scatterplot3d^64^, and corrgram^65^. Means ± SE are given throughout. All data are available from Mendeley Data (McGhee *et al*. 2020, 10.17632/rngtxkx9y4.1).

### Effect sizes from other studies

To compare our results to those of other studies, we estimated the effect sizes (Cohen’s *d* and *eta*^2^ ^66^) from published studies measuring glucocorticoids in individuals from groups differing in predation risk in the field. We restricted studies to those examining the influence of natural levels of predation risk in the field (i.e. studies with manipulations of risk were excluded), as well as those measuring hormones in unmanipulated adults (i.e. other manipulations such as food level, were excluded). Note that these measures are snapshots of glucocorticoid levels in the field and thus, could be due to acute short-term changes and/or long-term baseline differences. To estimate effect sizes, we used the means and standard deviations of concentrations of plasma cortisol/corticosterone or of fecal cortisol metabolite either extracted from the paper, estimated from the figures using GraphClick (version 3.0.3)^67^ or by contacting the authors directly. We then used G*Power^68^ to estimate the power of our design to detect similar effect sizes using an ANCOVA and a MANOVA.

## Results

**Figure 1:**
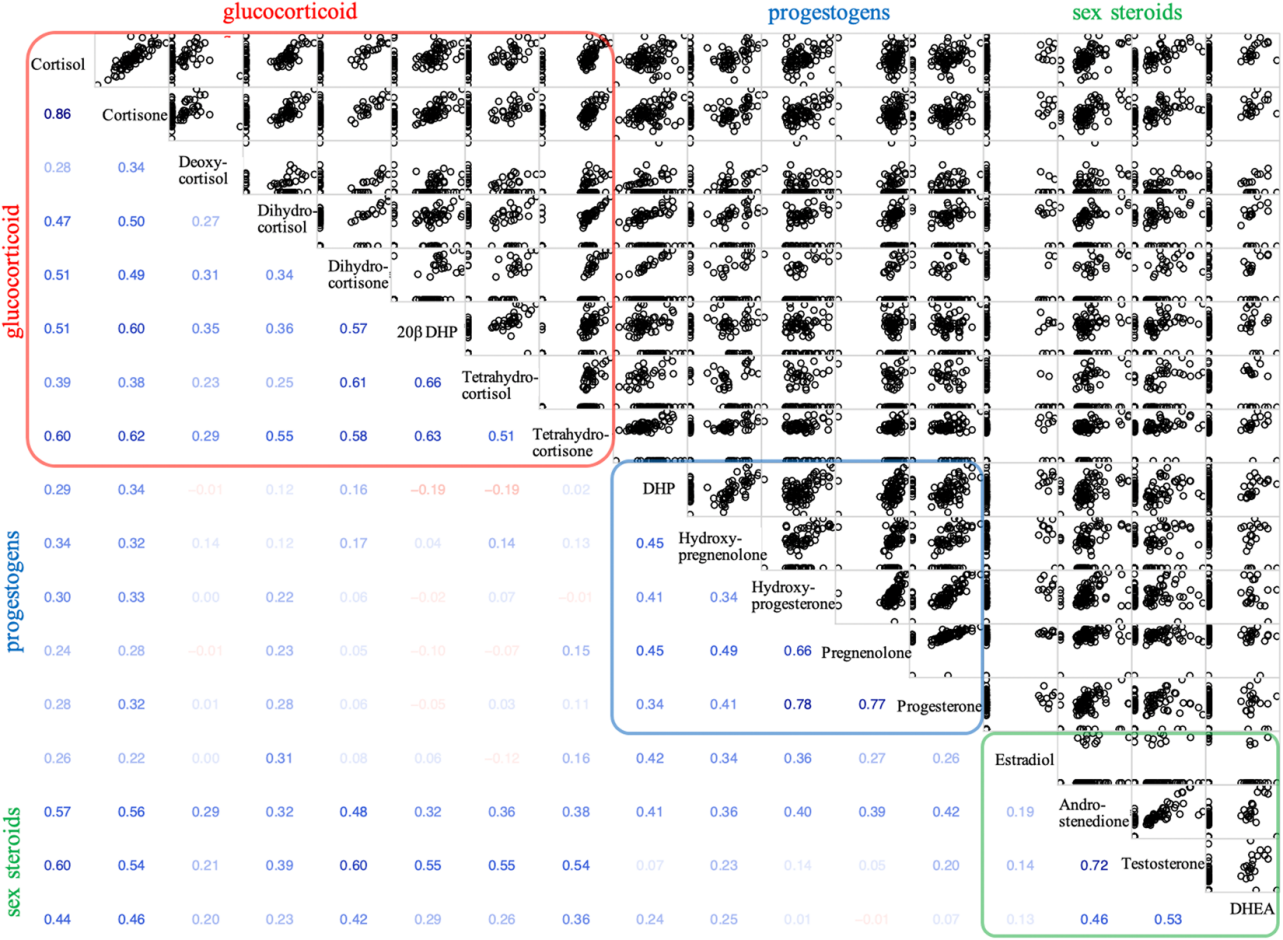
Correlations among the 17 hormones (standardized with the decostand method) for all predation regimes pooled (N = 89), with glucocorticoids, progestogens, and sex steroids highlighted in red, blue, and green respectively. Lower diagonal indicates the Spearman correlation coefficients with the shading and color showing the strength and direction (blue = positive, red = negative) of the correlations.

Of the 21 measured steroids, β-cortol was not included in the analyses because it was not detectable in any of the 89 clutches sampled. Similarly, etiocholanolone, ketotestosterone, and estrone were also removed from the analyses as they were rarely detected in the sampled clutches (number of clutches detected: etiocholanolone = 1, ketotestosterone = 2, estrone =5; see Table S3 for the number of clutches in which steroids were detected). Thus, we included 17 steroids in our analyses. Cortisol was detected at the highest levels across clutches (steroid means and standard errors are in Table S3), which is consistent with previous results in sticklebacks^69^. Steroid levels were variable among individuals and most steroids were positively associated with one another (Fig. 1). Some of the strongest associations were within classes (Fig. 1; maximum r_S_: glucocorticoids = 0.86, progestogens = 0.78, sex steroids = 0.72) but glucocorticoids also tended to be positively associated with androgens (maximum r_S_: 0.60). The relationships among glucocorticoids and progestogens (maximum r_S_: 0.34) and progestogens and sex steroids (maximum r_S_: 0.42) were weaker (Fig. 1).

We extracted three factors from the 17 steroid concentrations based on the parallel analysis and scree plot. Multiple steroids from the major steroid classes loaded strongly on separate factors resulting in factors that corresponded surprisingly well to separate steroid classes (Table 1). Steroids within a single class were not the only ones loading on a factor however, and steroids from other classes also loaded on each factor (Table 1). For example, Factor 1 comprised seven positively loaded glucocorticoids, two positively loaded androgens, and one negatively loaded progestogen (DHP). Factor 2 comprised five positively loaded progestogens as well as one estrogen (estradiol), and one glucocorticoid (cortisone). Factor 3 comprised three positively loaded sex steroids (three androgens), one progestogen (DHP) and four glucocorticoids. Most steroids loaded strongly on a single factor except for cortisol, cortisone, and androstenedione which loaded positively on all three factors. Additionally, the progestogen DHP loaded negatively on Factor 1 and positively on Factors 2 and 3. Testosterone and dihydrocortisone both loaded positively on Factors 1 and 3 (Table 1). Thus, although steroids within classes tended to co-vary, several steroids from different classes also played roles across more than one factor. For each steroid, the three factors together explained much of the variance within eggs (h2 in Table 1).

**Table 1:** Standardized rotated factor loadings of the wet concentrations of 17 steroids (ng/g) within clutches (N = 90 across 9 populations).

Steroid class	Measured steroids	Factor 1	Factor 2	Factor 3	h2 (communality)
Glucocorticoid	Cortisol	**0.54**	0.25	**0.63**	**0.74**
G	Cortisone	**0.55**	**0.34**	**0.62**	**0.81**
G	Deoxycortisol	0.09	−0.02	**0.31**	0.10
G	Dihydrocortisol	**0.51**	0.18	0.15	**0.32**
G	Dihydrocortisone	**0.55**	0.02	**0.45**	**0.51**
G	Tetrahydrocortisol	**0.69**	−0.01	0.13	**0.49**
G	Tetrahydrocortisone	**0.81**	−0.03	0.09	**0.67**
G	20β dihydrocortisone	**0.81**	−0.1	0.13	**0.68**
Progestogen	DHP	**−0.34**	**0.56**	**0.66**	**0.86**
P	HydroxyPregnenolone	0.07	**0.44**	0.28	0.28
P	HydroxyProgesterone	0	**0.84**	0.06	**0.71**
P	Pregnenolone	0	**0.56**	0.05	**0.32**
P	Progesterone	0.23	**0.87**	−0.15	**0.82**
Estrogen	Estradiol	−0.08	**0.38**	0.28	0.23
Androgen	Androstenedione	**0.37**	0.27	**0.60**	**0.70**
A	DHEA	0.26	0.04	**0.52**	**0.44**
A	Testosterone	**0.60**	0.06	**0.46**	**0.64**
SS Loadings (eigenvalues)		3.70	2.72	2.59	
Proportion variance (Cumulative variance)		0.22 (0.22)	0.16 (0.38)	0.15 (0.53)	RMSEA index: 0.12
Proportion explained (Cumulative explained)		0.41 (0.41)	0.30 (0.71)	0.29 (1.0)	

In bold are those steroids that have a loading of >0.3 on a particular factor, with bold and underlined values indicating loadings of >0.5. Bolded h2 values indicate those steroids where >30% of the variation in the steroid is explained by the 3 factors.

The CPC analysis suggests that the three predation regimes likely share three common principal components (all CPC vs 3CPC: AIC = 533.47; 3CPC vs 2CPC: AIC = 571.21; 2CPC vs 1CPC: AIC = 579.60; 1CPC vs unrelated: AIC = 597.60). The PCs (or factors) from the three regimes are unlikely to show equality or proportionality (Equality vs Proportional: AIC = 792.40; Proportional vs CPC: AIC = 743.54). Note that ‘Lake’ cannot be specified in these analyses, so we interpret these analyses with caution since we cannot account for the substantial variation among lakes.

**Figure 2:**
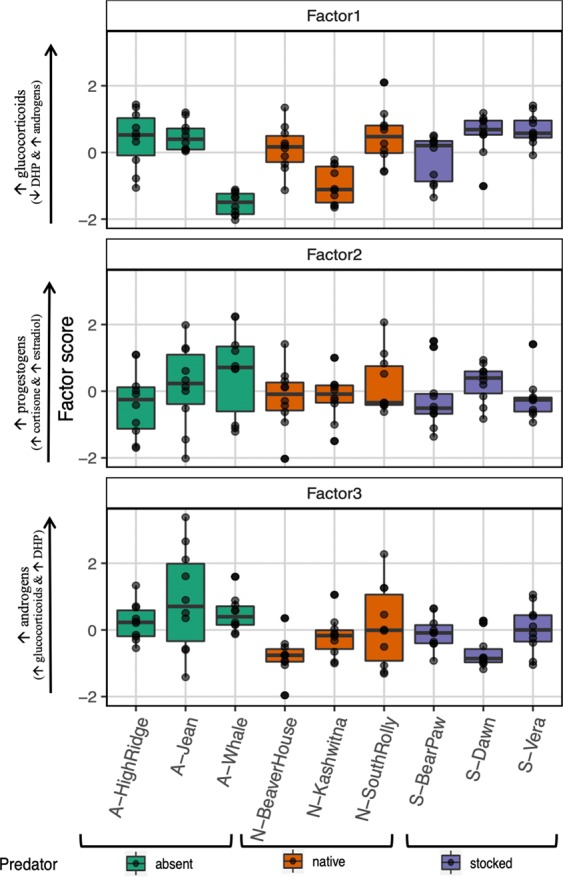
Boxplots indicating the rotated factor scores extracted from the wet concentrations of 17 different steroids among lakes without piscivorous fish (green: absent), with native piscivorous fish (orange: native) and with stocked piscivorous fish (purple: stocked) (N = 90 clutches, shown as black symbols; major steroid loadings (from Table 1) are indicated on the x-axis). Boxes enclose the interquartile range with the median indicated as a thick line and whiskers extending to 1.5X interquartile range.

**Figure 3:**
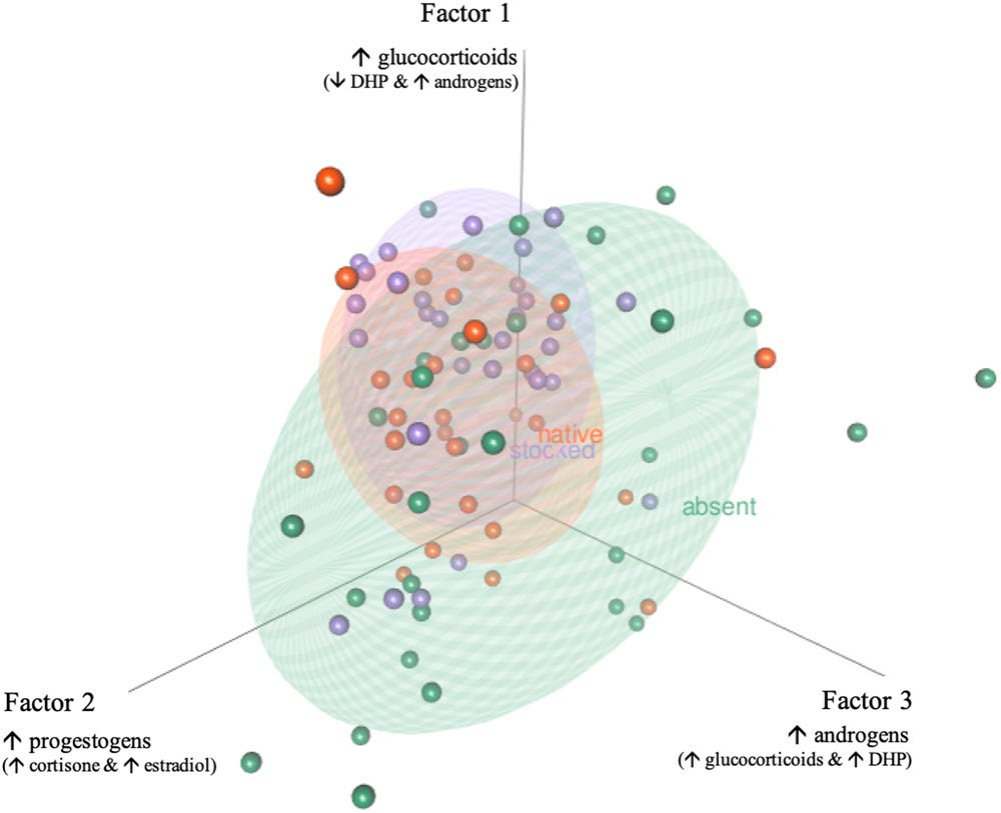
A three-dimensional plot illustrating the factor scores extracted from the wet concentrations of 17 different steroids for individuals in lakes either without piscivorous fish (green: absent), with native piscivorous fish (orange: native), or with stocked piscivorous fish (purple: stocked) with 95% ellipsoids (N = 90 clutches, shown as black symbols). Major steroid loadings, from Table 1, are indicated next to the axes.

**Figure 4:**
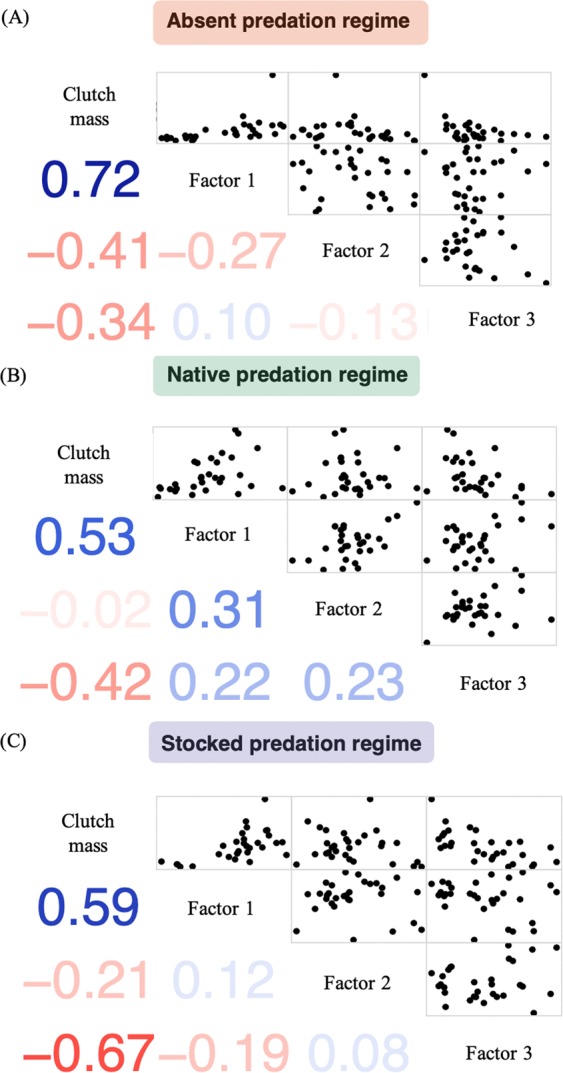
Correlations among the three extracted factors from the 17 hormones (from Table 1) and wet clutch mass (g) for (**A**) absent (N = 30), (**B**) native (N = 30), and (**C**) stocked predation regimes (N = 30). Values on the lower diagonal indicate the Spearman correlation coefficients with the shading and color showing the strength and direction (blue = positive, red = negative) of the correlations.

Although egg steroid profiles were variable among females and among lakes, we did not find a significant association between predator regime and the overall steroid profiles of eggs (Table 2a). Similarly, when the steroid profiles were summarized into three factors, predation regime did not significantly shape the dissimilarity matrix created from these steroid factors (Fig. 2, Table 2b). Restricting the factor analysis as well as the dissimilarity matrices to the reduced dataset (9 samples per lake; n = 81) instead of the dataset with the additional average sample for Whale Lake (10 samples per lake; n = 90) yields nearly identical results (Table S4). How these different steroid-factors were related to one another was also not associated with predation regime, and it is clear from the 3D plot, that the data from the different predation regimes overlaps entirely (Fig. 3). Steroid profiles were more variable among individuals from lakes without predators (absent lakes) compared to those with predators (native and stocked lakes) and variance within predation regimes (not accounting for the effect of lake) differed significantly for Factor 1 (chi-squared = 7.02, df = 2, P = 0.030) and Factor 2 (chi-squared = 8.74, df = 2, P = 0.013), but not Factor 3 (chi-squared = 0.51, df = 2, P = 0.773). Across all predation regimes, Factor 1 was positively associated with the wet mass of clutches (r_S_ = 0.65, P < 0.01, N = 90), while Factor 2 and 3 were both negatively associated with the wet mass of clutches (Factor 2: r_S_ = −0.23, P = 0.03; Factor 3: r_S_ = −0.54, P < 0.01; N = 90) (Fig. 4).

**Table 2:** Output from the permutational MANOVA examining whether Predator regime (absent, native, stocked) affects the dissimilarity matrices created on standardized data from the (a) wet concentrations of 17 different steroids within egg clutches (controlling for extraction efficiencies) and (b) the three factors extracted from the wet concentrations of these 17 steroids (in Table 1) (N = 90 clutches).

Factor	df	Sum of squares	Mean squares	F-value	R^2^	P-value
*(a) Dissimilarity matrix created from the 17 steroids:*						
Wet mass of clutch	1	372.0	372.0	5.140	0.047	0.003
Predator regime	2	301.7	150.8	2.084	0.038	0.805
Lake (Predator regime)	6	1511.7	251.9	3.481	0.189	0.402
Residuals	80	5790.6	72.4		0.726	
Total	89	7976.0			1.00	
*(b) Dissimilarity matrix created from the 3 extracted factors:*						
Wet mass of clutch	1	45.9	45.9	26.869	0.193	<0.001
Predator regime	2	11.5	5.7	3.363	0.048	0.680
Lake (Predator regime)	6	43.5	7.2	4.245	0.183	0.750
Residuals	80	136.6	1.7		0.575	
Total	89	237.6			1.00

**Figure 5:**
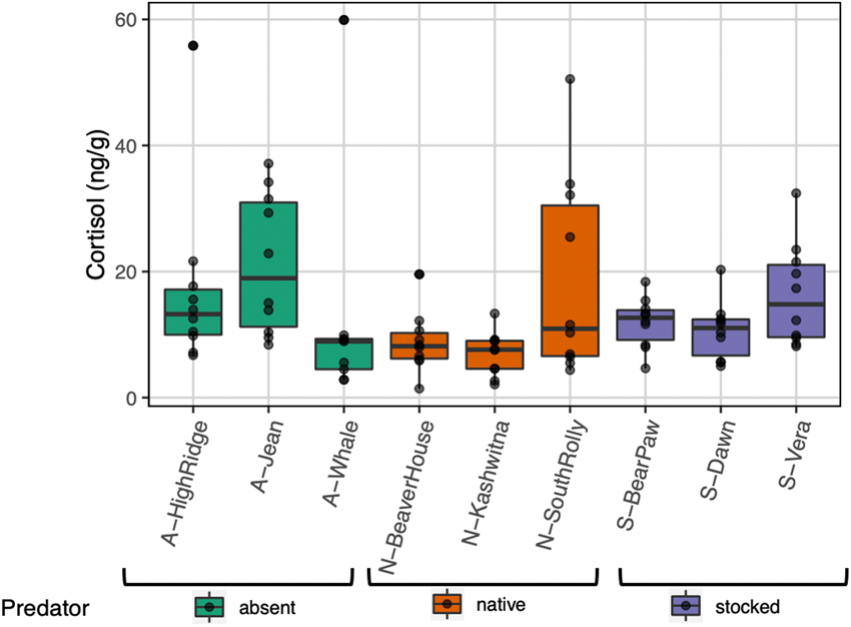
Boxplots showing the wet concentration of cortisol (ng/g; controlling for extraction efficiencies) among lakes without piscivorous fish (green: absent), with native piscivorous fish (orange: native) and with stocked piscivorous fish (purple: stocked) (N = 89 clutches, shown as black symbols). Boxes enclose the interquartile range with the median indicated as a thick line and whiskers extending to 1.5X interquartile range.

Despite being considered a major player in maternal stress, cortisol was not significantly associated with predation regime (Fig. 5; average concentration of cortisol ± SE: absent = 17.12 ± 2.68 ng/g, native = 11.49 ± 1.97 ng/g, stocked = 12.93 ± 1.12 ng/g; predation regime: F_2,5.68_ = 0.87, P = 0.465, wet clutch mass: F_1,84.97_ = 2.18, P = 0.143, random effect of lake: LRT = 7.26, P = 0.007, N = 89). Similarly, wet mass of clutch was not significantly associated with predation regime (average wet mass of clutch ± SE: absent = 0.165 ± 0.029 g, native = 0.173 ± 0.017 g, stocked = 0.233 ± 0.021 g; F_2,5.94_ = 0.84, P = 0.477, random effect of lake: LRT = 7.47, P = 0.006, N = 89). See Table S1 for population means and standard errors of cortisol and clutch mass.

We found 17 estimates from nine field studies of hormonal measures comparing either discrete years or populations that differed naturally in predation risk. There was substantial variation among these studies in the hormonal consequences of predation risk, with effect sizes ranging from *d* = 0.02 to *d* = 2.97 (Table 3). On average, individuals from high predation environments showed elevated glucocorticoid levels compared to individuals from low predation environments (Cohen’s *d* = 0.78 ± 0.18; Table 3). Our power in this study to detect a similar effect size in an ANCOVA analysis with three groups, a sample size of 30 samples per predation regime, and one covariate was 0.91. Furthermore, our power to detect an effect of *f*^2^ = 0.176 (with *f*^2^ = *eta*/(1 − *eta*)) using a MANOVA with a sample size of 90 and three groups was 0.78 for 20 measurements (i.e. hormones) and 0.99 for three measurements (i.e. factors). Thus we had sufficient power with our current dataset to detect the average effect size found in other studies. This again suggests that trout predation does not exert a consistently strong effect on the average steroid profiles of egg clutches in threespine stickleback from the field.

**Table 3:** Field estimates and effect sizes from previous studies of hormone levels between populations and years that differ naturally in predation risk^18,19,105–111^ (N=17 estimates).

Predator-Prey system		Hormones & Individuals Measured	High Predation			Low Predation			Effect sizes	
Prey	Predator		Identity	Mean (Std. dev.)	N	Identity	Mean (Std. dev.)	N	Cohen’s *d*	*eta*^*2*^
*Boonstra et al. (1998)*^106^^a^ *– Snowshoe hares in high (1991, 1992) vs low (1994) lynx abundance years*										
Snowshoe Hare	Lynx	Baseline plasma free cortisol (mmol/L):								
		Adults	1991	133.7 (269.22)	44	1994	42.2 (116.3)	36	0.43	0.044
		Adults	1992	117.4 (166.8)	36				0.52	0.063
*Clinchy et al. (2011)*^105^^ab^ *– Song sparrows breeding in mainland vs island populations*										
Song Sparrows	Terrestrial predators	Baseline plasma total corticosterone (nM/L):								
		Breeding female	Mainland	37.3 (28.1)	63^*b*^	Island	38.6 (23.1)	63^*b*^	0.05	0.001
		Breeding male	Mainland	52.8 (25.5)	49.5^*b*^	Island	38.0 (22.67)	49.5^*b*^	0.61	0.085
*Clinchy et al. (2004)*^107^^ab^ *– Song sparrows breeding in mainland vs island populations (both in unfed treatment)*										
Song Sparrows	Terrestrial predators	Maximum plasma corticosterone (ng/ml):								
		Breeding male	Mainland	116.6 (30.9)	11.5^*b*^	Island	90.4 (31.4)	11.5^*b*^	0.84	0.150
		Baseline plasma corticosterone (ng/ml)								
		Breeding male	Mainland	14.0 (3.93)	11.5^*b*^	Island	13.0 (4.9)	11.5^*b*^	0.22	0.012
*Graham et al. (2012)*^108^^a^ *– Fence lizards in fire ant invaded vs uninvaded populations*										
Fence Lizards	Fire Ants	Baseline plasma corticosterone (ng/ml):								
		Adult female	Invaded	23.7 (23.1)	18	Uninvaded	10.2 (9.8)	17	0.76	0.126
		Adult male	Invaded	5.8 (2.8)	12	Uninvaded	7.6 (10.8)	13	0.22	0.012
*Hammerschlag et al. (2017)*^19^^c^ *– Cape fur seals at high vs low shark abundance locations (both in high predation cold season)*										
Cape Fur Seals	White Sharks	Fecal glucocorticoid metabolite (ng/g):								
		Adults & juveniles	High sharks	1365201 (1049315)	102	Low sharks	847873 (1069374)	91	0.49	0.057
*Hik et al. (2001)*^109^ *– Arctic Ground Squirrels in alpine meadows vs boreal forest habitats*										
Arctic Ground Squirrel	Aerial & terrestrial predators	Baseline plasma free cortisol (nM/L):								
		Female	Boreal	219 (87.1)	10	Alpine	44.1 (16.8)	8	2.97	0.688
		Male	Boreal	124.7 (86.5)	10	Alpine	41.5 (9.2)	5	1.71	0.422
*Scheuerlein et al. (2001)*^110^^a^ *– Tropical stonechat breeding territories with vs without fiscal shrikes*										
Stonechats	Fiscal shrike	Baseline plasma corticosterone (ng/ml):								
		Females with fledged young	With shrikes	6.5 (10.5)	9	Without shrikes	6.3 (6.3)	9	0.02	0.0001
		Males with fledged young	With shrikes	21.0 (12.1)	9	Without shrikes	6.5 (6.6)	9	1.5	0.360
*Sheriff et al. (2010)*^111^ *– Snowshoe hares in high (2007) vs low (2006, 2008) lynx abundance years*										
Snowshoe Hare	Lynx	Fecal cortisol metabolite (ng/g):								
		Breeding females	2007	515.1 (142.9)	8	2006	352.2 (104.9)	10	1.32	0.303
						2008	430.3 (130.9)	10	0.62	0.088
*Sheriff et al. (2009)*^18^ *– Snowshoe hares in high (2007) vs low (2006, 2008) lynx abundance years*										
Snowshoe Hare	Lynx	Fecal cortisol metabolite (ng/g):								
		Breeding females	2007	476.6 (356.5)	11	2006	383.5 (383.1)	10	0.25	0.015
						2008	263.9 (168.9)	9	0.74	0.120
							***AVERAGE***		**0.78**	**0.150**
							***Standard error***		**0.18**	**0.046**

^a^Means and standard deviations extracted from figure using GraphClick.

^b^Sample sizes unclear and were averaged from the maximum n provided in paper.

^c^Means and sample sizes obtained from authors.

## Discussion

We found that the 17 maternally-derived steroids detected in eggs primarily clustered together within classes (glucocorticoids, progestogens, and sex steroids) with particular steroids from each class loading strongly on more than one factor. It is important to note that the LC-MS/MS method used here measures the steroids precisely, thus covariation among steroids is not due to antibody cross reactivity (which can be a problem with other methods). While these results emphasize the modular yet interconnected nature of the endocrine response, surprisingly predation regime (absent, native, stocked) was not associated with these maternally-derived steroid egg profiles.

Our results demonstrate that many different maternally-derived steroids are detectable in unfertilized fish eggs and these steroids tend to positively covary and change in concert. Furthermore, particular steroids (e.g. cortisol, cortisone, dihydrocortisone, DHP, Testosterone) contribute strongly to multiple factors suggesting that they might be more influential to embryonic development than others. Most studies on the intergenerational effects of maternal stress, especially in egg laying vertebrates, have used exogenous manipulations of cortisol/corticosterone in mothers or in their eggs to mimic maternal stress^28,34,35,37^. While there is little doubt that the production of glucocorticoids in mothers is vital to how maternal stress effects are produced, changes in any or all of the maternally-derived steroids (as well as other components, such as miRNAs) in eggs could serve as effective signals of maternal stress. For example, maternal glucocorticoids, progestogens, androgens, and estrogens, have all been shown to influence offspring development in egg laying vertebrates^52,70^. Additionally, developmental effects can occur without direct exposure of offspring to maternal glucocorticoids^38,39^. For example, stickleback embryos are able to buffer themselves from exogenous manipulations of cortisol by actively transporting cortisol out of the egg via ATP-binding cassette transporters^69,71^. This suggests that the effects of maternal predator exposure on offspring behavior^72–74^, physiology^75^ and embryonic gene transcription^76^ in threespine stickleback are not mediated solely by embryonic exposure to maternal cortisol. Our results showing that many progestogens and androgens are positively correlated with glucocorticoids further emphasize the interconnected nature of the endocrine system and suggest that some assumptions, such as the negative relationship between androgens and glucocorticoids, might not be detectable in the maternally-derived steroids in eggs. Acknowledging that many maternal steroids play a role in offspring development and can vary in a coordinated fashion within eggs, will help us move beyond the focus on maternally derived glucocorticoid steroids^2,38,77^.

That we did not see a consistent pattern of predation regime on steroid profiles was unexpected (but see^78^), particularly given the role of glucocorticoids in dealing with predator encounters^2,14,79^, as well as the findings that stress-induced cortisol levels can be sensitive to rearing conditions^80^, heritable^80,81^, and respond to selection^82^. Not only is there no obvious difference between lakes with and without predatory trout, but exposure to piscine predators for 25 or fewer generations (stocked lakes) and ~6,000 generations (native lakes) have similar consequences for the steroid profiles. Although we do not have phenotypic measures of adults from our Alaskan populations, multiple studies have demonstrated parallel evolution of a variety of morphological traits in response to predation regime^6^. For example, fish from high predation populations differ in body shape and armor compared to low predation populations^48,83,84^, with these differences arising in fewer than 10 years in stickleback^44^. Additionally, our review of field studies suggests that population-level predation risk should be detectable in snapshots of circulating glucocorticoids. We had assumed based on this and the physiological consequences of predator encounters^16^ that adult females from these populations would differ in their circulating glucocorticoids (at least) and this would be reflected in their eggs. Sampling steroids of adults from these populations is clearly an avenue for future research but our results suggest that there is not a strong pattern between the predation risk experienced by mothers and the maternally-derived steroids in eggs.

Interestingly, we did find that stocked and native populations show less variation in both Factors 1 and 2 of their steroid profiles compared to populations in which predation is absent. It is possible that predators might exert stabilizing selection on prey and select for less variable and potentially more fine-tuned responses from prey. Mounting a stress response to each predator sighting in a high-predation environment can result in deleterious health consequences^13,24,85^. In order to cope with the chronic stress of repeated predator encounters, there is evidence that sensitivity to predator exposure and stress responsiveness might decrease, either as an evolved response^11,86^ or as a plastic response due to habituation^85^, or stress inoculation^87,88^. For example, high-predation risk populations tend to show lower levels of cortisol after a stressor compared to low predation risk populations^21,22^. As potent mediators of embryonic development^36^, cortisol and other maternally-derived hormones should be tightly regulated to prevent developmental disruptions^31^. Thus, it is possible that there has been strong selection for mechanisms that reduce the maternal stress response, or the downstream effects of maternal stress, resulting in the absence of any strong signature of predation risk in eggs (countergradient variation or cryptic evolution^89^). Disentangling the roles of plasticity and genetic effects in these patterns with the use of common garden studies over multiple generations and a reaction norm approach^77^ is an interesting area of future study.

How the steroid profiles measured here correspond to the phenotype under selection is unknown and different combinations of steroids might result in similar phenotypes with equivalent fitness (many-to-one mapping of form to function^5,90^). This “many-to-one” hypothesis has been proposed as an explanation for the inconsistencies between the strong patterns of parallelism in studies of morphological traits and the absence of such parallelism in studies of biomechanical and physiological traits as well as studies of the underlying genetics^6,91–94^ (but see^95^). The “many-to-one” hypothesis could be particularly relevant for steroids because of their role in phenotypic integration and coordinating suites of traits^96,97^. Interestingly, recent modeling efforts of hormonal pleiotropy has found that although the shapes of hormonally controlled resource allocation trade-offs show phenotypic convergence, they are often underlain by a variety of physiological mechanisms (i.e. different genetic solutions to the same problem)^98^. How steroid profiles translate into phenotypes and whether different combinations of maternally-derived steroids can give rise to similar offspring phenotypes with equal performance is clearly an area for future research.

The endocrine response is necessarily highly plastic (reviewed in^14,24^). Our review of field studies suggests that although predation risk is, in general, associated with increased glucocorticoid levels in prey, it is also highly variable in the strength of its effects. Some of the variation within high predation populations in steroid profiles, including our native and stocked predation regime lakes, could be due to variation among individuals in their personal experience with predators^23^. For example, steroids can vary with how recently, or how often, individuals have encountered a predator^16,17^. How females respond to predators may also have indirect consequences manifested as changes in foraging behavior and habitat use^2,99^ with such changes affecting female steroid levels^1^. Furthermore, experiences with predators are not the only factors capable of affecting the endocrine system. For example, during the mating season, gravid females interact with multiple courting and potentially aggressive males, they associate and observe other females, and they make foraging decisions and compete for resources in order to invest in egg production. The social environment alone is likely to vary within and among populations, may or may not be associated with predation regime, and can affect hormone levels^100^. Although this sensitivity to the external environment suggests that endocrine phenotypes might be more variable than other traits, corticosterone measures exhibit similar levels of variability to other phenotypic measures among populations^101^ and are generally repeatable across studies (r = 0.29^102^). Ecological similarities across lakes that are unrelated to predation regime might offer an explanation for the dominance of particular steroids across multiple factors as well as the similar factor loadings across regimes (CPC results). How plasticity due to individual experiences (to predators, mates, competitors) interacts with the background risk of predation in a population to shape an individual’s steroid profile is unknown.

Another outstanding question concerns how and when maternal steroids influence the steroid environment experienced by developing offspring because few studies have attempted to link maternal steroids in ovaries or in circulation to steroids in eggs, embryos and/or later in offspring development in fishes^28,103,104^. Whether steroid content in embryos reflects recent/current maternal history or the mother’s more distant past^103,104^ is not well understood. There is some evidence that cortisol in fish embryos is reflective of at least several days of past maternal steroid levels and potentially maternal steroids over the entire yolking process (i.e. the exposure of vitellogenic oocytes)^103^. For example, daily consumption of cortisol-spiked food was detectable in maternal ovaries after 5 days as well as transiently in embryos 3 days later in zebrafish^103^. Work on field-collected adult females from California stickleback populations suggests that being chased briefly (30 sec per day) by a model predator over the course of ~one month in the laboratory is enough to override past experiences and be detectable in embryo cortisol levels^72^, and influence offspring behavior^74^. That being said, it is important to keep in mind that accumulation of steroids in embryos is also not a passive process; *in vitro* exposure of fish ovarian follicles to elevated cortisol suggests that upregulation of corticosteroid silencing genes protects embryos from excess maternal cortisol^103^ and embryos themselves can actively remove maternal cortisol from the egg^71^. It is clear that substantially more work is needed to mechanistically link maternal steroid profiles to those of their eggs, as well as to elucidate the timescale over which maternal experiences are reflected in maternally-derived egg steroid profiles.

Using methodology that allowed us to quantify multiple steroids simultaneously in eggs, we examined for the first time whether the maternally-derived steroids in eggs correspond to predation regime across different populations. Our results emphasize the coordinated and interconnected fashion in which maternally-derived steroids vary within and among steroid classes. Furthermore, our results caution against assuming predation risk is detectable in steroid profiles of eggs in the field. Predator encounters may indeed affect maternal stress and thus, embryonic exposure to maternal steroids, but this pattern is clearly more complex than a simple one-to-one relationship between predation risk at the population level and the multiple steroids mothers deposit in their eggs.

## Supplementary information

Supplementary information.

## Data Availability

Data available from Mendeley Data (McGhee *et al*. 2020, 10.17632/rngtxkx9y4.1).
