# Supplementary material for: Effects of predation risk on egg steroid profiles across multiple populations of threespine stickleback
Source: Sci Rep. 2020 Mar 23;10:5239. doi: 10.1038/s41598-020-61412-5 (PMC7090078; doi:10.1038/s41598-020-61412-5)
Supplement: Supplementary file 1 — Supplementary information. [file 41598_2020_61412_MOESM1_ESM.docx]

Title: Effects of predation risk on egg steroid profiles across multiple populations of threespine stickleback

Authors: Katie E. McGhee, Ryan T. Paitz, John A. Baker, Susan A. Foster, and Alison M. Bell

SUPPLEMENTARY MATERIALS

| Population | Predation Regime | Initial trout stocking | Latitude | Longitude | Approx. elevation (m) | Approx. surface area (ha) | N clutches | Mean (SE) clutch wet mass (g) | Mean (SE)  cortisol wet concentration (ng/g) |
| --- | --- | --- | --- | --- | --- | --- | --- | --- | --- |
| High Ridge | Absent | - | 61.5783 | -149.1778 | 27 | 15.9 | 10 | 0.183 (0.019) | 17.140 (4.543) |
| Jean | Absent | - | 61.7264 | -150.0639 | 76 | 21.8 | 10 | 0.237 (0.076) | 21.217 (3.507) |
| Whale | Absent | - | 61.5419 | -149.7516 | 70 | 5.1 | 9 | 0.065 (0.008) | 12.555 (5.994) |
| Beaver House | Native | - | 61.5736 | -149.8625 | 52 | 17.0 | 10 | 0.230 (0.0285) | 8.782 (1.517) |
| Kashwitna | Native | - | 61.8333 | -150.0764 | 56 | 65.2 | 10 | 0.106 (0.009) | 6.973 (1.098) |
| South Rolly | Native | - | 61.6679 | -150.1391 | 58 | 43.7 | 10 | 0.182 (0.031) | 18.718 (5.011) |
| Bear Paw | Stocked | 1992 | 61.6139 | -149.7756 | 82 | 16.0 | 10 | 0.169 (0.033) | 11.911 (1.262) |
| Dawn | Stocked | 1992 | 61.5450 | -149.7000 | 84 | 4.8 | 10 | 0.0441 (0.041) | 0.268 (1.452) |
| Vera | Stocked | 1992 | 61.7125 | -150.1375 | 61 | 44.9 | 10 | 0.236 (0.025) | 16.272 (2.544) |

Table S1. Location and background information about the nine Alaska populations as well as average (+/- SE) clutch wet mass and cortisol values (wet concentration, ng/g) for the clutches.

Table S2. The 21 steroids quantified in stickleback eggs using LC/MS/MS along with the multiple-reaction-monitoring transitions, retention times used for their quantification, extraction efficiencies (% recovery) and coefficients of variation. Steroid classes as follows: P=Progestogen, G=Glucocorticoid, A=Androgen, E=Estrogen

| **Common Name**  **(Steroid)** | **Steroid Class** | **Chemical**  **Formula** | **MW** | **CAS #** | **Q1**  **(m/z)** | **Q3 (m/z)** | **Retention Time** | **Recovery** | **CV** |
| --- | --- | --- | --- | --- | --- | --- | --- | --- | --- |
| DHP Dihydroprogesterone  (4-PREGNEN-17, 20β-DIOL-3-ONE) | P | C21 H32 O3 | 332.48 | 1662-06-2 | 333 | 97 | 17.1 | 0.6 | 9.6 |
| 17α-hydroxypregnenolone  (5-PREGNEN-3β, 17-DIOL-20-ONE) | P | C21 H32 O3 | 332.48 | 387-79-1 | 333 | 297 | 18.0 | 0.672675 | 6.3 |
| Progesterone  (4-PREGNEN-3, 20-DIONE) | P | C21 H30 O2 | 314.46 | 57-83-0 | 315 | 97 | 22.3 | 0.708402 | 9.9 |
| Pregnenolone  (5-PREGNEN-3β-OL-20-ONE) | P | C21 H32 O2 | 316.48 | 145-13-1 | 317 | 299 | 21.7 | 0.660861 | 10.1 |
| 17α-hydroxyprogesterone  (4-PREGNEN-17-OL-3, 20-DIONE) | P | C21 H30 O3 | 330.46 | 68-96-2 | 331 | 97 | 18.9 | 0.664208 | 6.3 |
| Cortisone  (4-PREGNEN-17, 21-DIOL-3, 11, 20-TRIONE) | G | C21 H28 O5 | 360.44 | 53-06-5 | 361 | 163 | 11.7 | 0.606363 | 12.6 |
| 20β-dihydrocortisone  (4-PREGNEN-17, 20β, 21-TRIOL-3, 11-DIONE) | G | C21 H30 O5 | 362.46 | 116-59-6 | 363 | 163 | 9.4 | 0.829007 | 5.5 |
| Cortisol  (4-PREGNEN-11β, 17, 21-TRIOL-3, 20-DIONE) | G | C21 H30 O5 | 362.46 | 50-23-7 | 363 | 121 | 10.9 | 0.666571 | 8.6 |
| 5β-dihydrocortisol  (5β-PREGNAN-11β, 17, 21-TRIOL-3, 20-DIONE) | G | C21 H32 O5 | 364.48 | 1482-50-4 | 365 | 335 | 13.6 | 0.722454 | 8.5 |
| 5β-tetrahydrocortisol  (5β-PREGNAN-3α, 11β, 17, 21-TETROL-20-ONE) | G | C21 H34 O5 | 366.49 | 53-02-01 | 367 | 331 | 11.7 | 0.6195 | 11.1 |
| β-cortol  (5β-PREGNAN-3α, 11β, 17, 20β, 21-PENTOL) | G | C21 H36 O5 | 368.51 | 667-65-2 | 369 | 333 | 11.8 | 0.570623 | 8.8 |
| 5β-dihydrocortisone  (5β-PREGNAN-17, 21-DIOL-3, 11, 20-TRIONE) | G | C21 H30 O5 | 362.46 | 68-54-2 | 363 | 315 | 13.6 | 0.736775 | 9.4 |
| 5β-tetrahydryocortisone  (5β-PREGNAN-3α, 17, 21-TRIOL-11, 20-DIONE) | G | C21 H32 O5 | 364.48 | 53-05-4 | 365 | 347 | 13.5 | 0.695343 | 6.5 |
| 11-deoxycortisol  (4-PREGNEN-17, 21-DIOL-3, 20-DIONE) | G | C21 H30 O4 | 346.46 | 152-58-9 | 347 | 97 | 15.0 | 0.734661 | 7.9 |
| 11-ketotestosterone  (4-ANDROSTEN-17β-OL-3, 11-DIONE) | A | C19 H26 O3 | 302.41 | 564-35-2 | 303 | 121 | 12.9 | 0.748121 | 5.6 |
| DHEA  (5-ANDROSTEN-3β-OL-17-ONE) | A | C19 H28 O2 | 288.42 | 53-43-0 | 289 | 253 | 18.5 | 0.628757 | 11.9 |
| Etiocholanolone  (5β-ANDROSTAN-3α-OL-17-ONE) | A | C19 H30 O2 | 290.44 | 53-42-9 | 291 | 255 | 20.2 | 0.582732 | 6.6 |
| Testosterone  (4-ANDROSTEN-17β-OL-3-ONE) | A | C19 H28 O2 | 288.42 | 58-22-0 | 289 | 97 | 17.2 | 0.648449 | 11.1 |
| Androstenedione  (4-ANDROSTEN-3, 17-DIONE) | A | C19 H26 O2 | 286.41 | 63-05-8 | 287 | 97 | 18.8 | 0.645356 | 14.4 |
| Estrone  (1, 3, 5(10)-ESTRATRIEN-3-OL-17-ONE) | E | C18 H22 O2 | 270.37 | 53-16-7 | 271 | 253 | 19.4 | 0.627634 | 5.2 |
| Estradiol  (1, 3, 5(10)-ESTRATRIEN-3, 17β-DIOL) | E | C18 H24 O2 | 272.38 | 50-28-2 | 273 | 107 | 17.6 | 0.843082 | 6.8 |

Table S3. Mean and standard errors of wet concentrations of the 21 steroids (ng/g) examined in stickleback eggs (N=89) correcting for the extraction efficiencies. Steroid classes as follows: P=Progestogen, G=Glucocorticoid, A=Androgen, E=Estrogen

| **Common Name** | **Steroid Class** | **Mean Wet Concentration** | **WC Standard error** | **Number of samples where detectable** |
| --- | --- | --- | --- | --- |
| DHP (Dihydroprogesterone) | P | 2.893075 | 0.262771 | 89 |
| 17α-hydroxypregnenolone | P | 5.007541 | 1.052226 | 57 |
| Progesterone | P | 0.409062 | 0.049438 | 78 |
| Pregnenolone | P | 8.238251 | 0.556236 | 87 |
| 17α-hydroxyprogesterone | P | 8.712956 | 2.1184 | 88 |
| Cortisone | G | 3.427235 | 0.257953 | 89 |
| 20β-dihydrocortisone | G | 0.314886 | 0.080302 | 59 |
| Cortisol | G | 13.81169 | 1.178465 | 89 |
| 5β-dihydrocortisol | G | 0.225597 | 0.031473 | 49 |
| 5β-tetrahydrocortisol | G | 0.236205 | 0.049717 | 33 |
| 5β-dihydrocortisone | G | 0.314886 | 0.080302 | 22 |
| 5β-tetrahydryocortisone | G | 2.09002 | 0.266754 | 66 |
| β-cortol | G | 0 | 0 | 0 |
| 11-deoxycortisol | G | 0.808525 | 0.711911 | 30 |
| 11-ketotestosterone | A | 0.005026 | 0.003635 | 2 |
| DHEA | A | 1.07472 | 0.387687 | 21 |
| Etiocholanolone | A | 0.005645 | 0.005645 | 1 |
| Testosterone | A | 1.869337 | 0.796831 | 52 |
| Androstenedione | A | 2.144293 | 0.753543 | 84 |
| Estrone | E | 0.553516 | 0.271442 | 5 |
| Estradiol | E | 1.155722 | 0.390227 | 10 |

Table S4. Output from the permutational MANOVA examining whether Predator regime (absent, native, stocked) affects the dissimilarity matrices created on standardized data from the (a) wet concentrations of 17 different steroids within egg clutches and (b) the three extracted factors (in Table 1) with one random data point removed from each lake (except Whale Lake). P-values were estimated with 10000 permutations specifying that clutches were permutated within Lake and Lake within Predation regime (N=81 clutches total from 9 females per lake).

| **Factor** | **df** | **Sum of squares** | **Mean**  **squares** | **F-value** | **R^2^** | **P-value** |
| --- | --- | --- | --- | --- | --- | --- |
| *(a) Dissimilarity matrix created from the 17 steroids (n=81):* | | | | | | |
| Wet mass of clutch | 1 | 331.3 | 331.3 | 4.667 | 0.048 | 0.005 |
| Predator regime | 2 | 250.4 | 125.2 | 1.764 | 0.036 | 0.819 |
| Lake (Predator regime) | 6 | 1328.4 | 221.4 | 3.119 | 0.191 | 0.348 |
| Residuals | 71 | 5040.2 | 70.99 |  | 0.725 |  |
| Total | 80 | 6950.2 |  |  | 1.00 |  |
| *(b) Dissimilarity matrix created from the 3 extracted factors (n=81):* | | | | | | |
| Wet mass of clutch | 1 | 39.4 | 39.4 | 20.365 | 0.175 | <0.001 |
| Predator regime | 2 | 12.1 | 6.0 | 3.127 | 0.054 | 0.557 |
| Lake (Predator regime) | 6 | 36.7 | 6.1 | 3.162 | 0.163 | 0.817 |
| Residuals | 71 | 137.3 | 1.9 |  | 0.609 |  |
| Total | 80 | 225.5 |  |  | 1.00 |  |
